# Dynamics of the Metabolome of *Aliinostoc* sp. PMC 882.14 in Response to Light and Temperature Variations

**DOI:** 10.3390/metabo11110745

**Published:** 2021-10-29

**Authors:** Damien Le Moigne, Justine Demay, Anita Reinhardt, Cécile Bernard, Sandra Kim Tiam, Benjamin Marie

**Affiliations:** 1 UMR7245 Molécules de Communication et Adaptation des Micro-Organismes (MCAM) MNHN-CNRS, Muséum National d’Histoire Naturelle, CP 39, 12 Rue Buffon, CEDEX 05, F-75231 Paris, France; Damien.LeMoigne@cea.fr (D.L.M.); justine.demay1@mnhn.fr (J.D.); cecile.bernard@mnhn.fr (C.B.); sandra.kim-tiam-fook-chong@univ-lyon1.fr (S.K.T.); 2 Thermes de Balaruc-Les-Bains, 1 Rue du Mont Saint-Clair BP 45, 34540 Balaruc-Les-Bains, France; anita.reinhardt@thermesbalaruc.com; 3 UMR5557 Laboratoire d’Ecologie Microbienne, Université de Lyon, 43 bd du 11 novembre 1918, Villeurbanne, F-69622 Lyon, France

**Keywords:** cyanobacteria, metabolomics, high-resolution mass spectrometry, secondary metabolite induction, culture conditions

## Abstract

Cyanobacteria are microorganisms able to adapt to a wide variety of environmental conditions and abiotic stresses. They produce a large number of metabolites that can participate in the dynamic adaptation of cyanobacteria to a range of different light, temperature, and nutrient conditions. Studying the metabolite profile is one way to understand how the physiological status of cells is related to their adaptive response. In this study, we sought to understand how the diversity and dynamics of the whole metabolome depended on the growth phase and various abiotic factors such as light intensity and temperature. The cyanobacterium, *Aliinostoc* sp. PMC 882.14, was selected for its large number of biosynthetic gene clusters. One group of cells was grown under normal conditions as a control, while other groups were grown under higher light or temperature. Metabolomes were analyzed by mass spectrometry (qTOF-MS/MS) combined with untargeted analysis to investigate metabolite dynamics, and significant variation was found between exponential and stationary phases, regardless of culture conditions. In the higher light group, the synthesis of several metabolites, including shinorine, was induced while other metabolites, such as microviridins, were synthesized under higher temperature conditions. Among highly regulated metabolites, we observed the presence of mycosporine-like amino acids (MAAs) and variants of somamides, microginins, and microviridins. This study demonstrated the importance of considering the physiological state of cyanobacteria for comparative global metabolomics and studies of the regulatory processes involved in production of specific metabolites. Our results also open up new perspectives on the use of organisms such as cyanobacteria for the targeted production of bioactive metabolites.

## 1. Introduction

Cyanobacteria are Gram-negative photosynthetic prokaryotes that can be found in all environments and habitats including extreme conditions. They play an essential role in the functioning of various ecosystems because of their involvement in primary production through oxygenic photosynthesis. They have been shown to contain the pigments phycocyanin and phycoerythrin, in addition to chlorophyll a [1]. Cyanobacteria produce a wide variety of bioactive metabolites, more than 1100 of which have been described to date [2]. This array of metabolites seems to support the remarkable ecological capacities of cyanobacteria [3]. As a consequence, cyanobacteria are the organisms of choice for researching methods for production of valuable bioactive molecules. Scientific fields such as pharmacology, the food industry, and biotechnology are actively exploring their properties for potential commercialization [4,5]. Cyanobacteria also present promising applications in the field of cosmetics thanks to their production of various molecules with photo-protective, antioxidant, or anti-inflammatory properties, such as carotenoids, mycosporine-like amino acids (MAAs), and scytonemins [5,6,7].

Many of the diverse cyanobacterial secondary metabolites are peptides or exhibit peptide-like structures [8]. They are synthesized via specific enzymatic pathways [2] through either ribosomal (ribosomally synthesized and post-translationally modified peptides: RiPPs) or non-ribosomal (non-ribosomal peptide synthase, NRPS, or polyketide synthase, PKS) pathways [9]. Other important secondary metabolites can present different structural organizations that belong to chemical classes such as alkaloids, terpenes, polysaccharides, or even lipids [10]. Despite many decades of work, the biological roles of the vast majority of these cyanobacterial secondary metabolites remain unknown [11]. A number of hypotheses regarding their respective potential biological functions (e.g., allelopathy, defense, parasitism/symbiosis, chemical communication, etc.) have been proposed [12].

There have been a relatively large number of studies performed on the effects of physiological status or growth phase on the production of targeted molecules in vitro [13], but only a few of these have been focused on the whole cyanobacterial metabolome and its dynamics over time and under different culture conditions [14]. Interestingly, a pilot study performed on diatoms [15] demonstrated a clear dependence of metabolic profiles on different growth phases: exponential, stationary, and senescent. Several factors are thought to influence the growth of cyanobacteria, including temperature, light, and nutrient availability. Some researchers have shown that a temperature of 25 °C improved the growth of bloom-forming Microcystis strains while a lack of light or nutrients negatively impacted the growth of cyanobacteria [16,17,18]. Beyond its effects on cyanobacterial growth, light has also been described as a key factor driving the production of specific metabolites. For example, cyanobacteria can activate photoprotection mechanisms by synthesizing molecules such as MAAs that absorb ultraviolet radiation [19]; other systems minimize the production of ROS and protect DNA thanks to specific UV-inducible defenses such as scytonemins [6].

**Figure 1:**
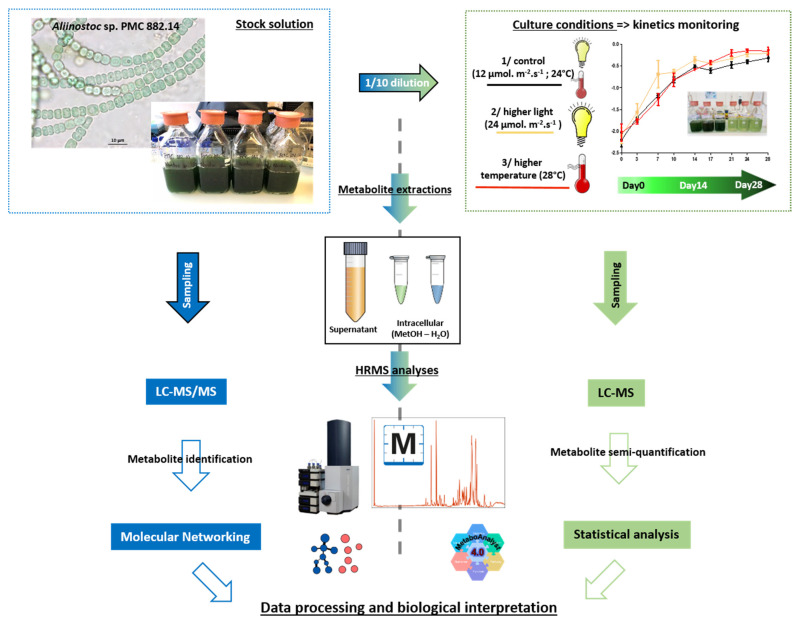
Graphical abstract of the analytical pipeline performed in this study.

In the present study, we investigated the entire metabolite profile produced by the cyanobacterium, *Aliinostoc* sp. PMC 882.14, isolated from biofilms on the thermal mud of Balaruc-les-Bains (France) [20].(Figure 1) *Aliinostoc* sp. PMC 882.14 was selected because it exhibits a large number of biosynthetic gene clusters (BGCs) (Table S1) able to produce a wide variety of metabolites. Many of these are valuable in the cosmetic or pharmaceutical sectors because of their antioxidant and UV-protective properties (MAAs) or their cytotoxic activity (microginin, symplostatin, and dolastatin) (Figure S1) [21].

This work describes the diversity of metabolites produced by *Aliinostoc* sp. PMC 882.14 and illustrates the importance of culture conditions on the metabolite content using qualitative and quantitative untargeted metabolomic approaches. We focused on the effects of light and temperature on the composition of endo- and exo-metabolites along with cyanobacterial growth. We hypothesized that increasing light intensity and temperature increased the production of specific metabolites, allowing adaptation of cyanobacteria to variations in these two abiotic factors. We characterized some of them with regard to their relevance for cosmetic or pharmaceutical applications.

## 2. Results

### 2.1. Extraction Compartment Specificity and Metabolite Diversity of Aliinostoc sp. PMC 882.14

The three fractions first investigated using both positive and negative ionization MS modes included (1) intracellular metabolites extracted with water (IW), (2) intra-cellular metabolites extracted with methanol (IM), and (3) metabolites from the extra-cellular supernatants (E) of *Aliinostoc* sp. PMC 882.14 cultured first under normal control conditions. From these three fractions, 1971 different ions were observed in positive mode (79% of total ions detected), while 518 ions (20% of the total ions detected) were observed in negative mode, with only 48 ions detected in common in both positive and negative ionization modes. Accordingly, we chose to focus our subsequent metabolome investigation on the results obtained from MS analysis performed on the positive mode.

**Figure 2:**
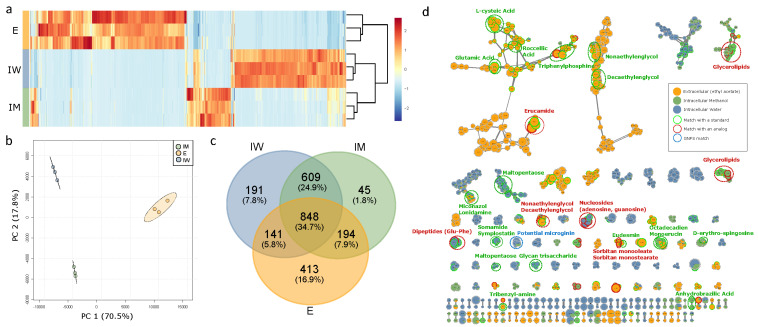
Metabolic composition of the three extraction fractions (triplicates) illustrated by (**a**) a heatmap with hierarchical classification, (**b**) a principal component analysis, (**c**) a Venn diagram, and (**d**) a molecular network representation of the molecules detected in each one of the three extraction fractions. E, extracellular; IW, intracellular-water; IM, intracellular-methanol. On the molecular network, the dots represented the different ions analyzed in autoMS/MS (merged positive and negative mode dataset). The colors of the dots corresponded to the fraction in which the analytes have been detected. The annotations were made with the MetGem 1.2.1 software (red and green dotted circles) or with the GNPS web tool (blue dotted circle) using public spectral databases. Only clusters of at least two nodes are represented.

Figure 2 exhibits the respective composition (in relative intensity) of the ions analyzed by LC-MS (using merged positive and negative mode datasets) for the three fractions analyzed (IW, IM, and E). Figure 2a shows that the three extraction fractions have a very different metabolic composition, especially the extracellular fraction, E, that presents a very different metabolic composition from the two intracellular fractions, IW and IM. These observations were also illustrated on the individual plots of principal component analysis (PCA; Figure 2b) and the Venn diagram (Figure 2c), indicating that similar numbers of analytes (chemical entities representing all observed charge states and adduct forms) were observed in the three fractions (totals of 1596 in E, 1789 in IM, and 1693 analytes detected in the IW fraction), with 848 analytes (34.7%) being present in all three extracted fractions. The E fraction contained 413 unique analytes (16.9% of the total analytes detected), while fraction IW had 191 (7.8%) and fraction IM had 45 unique ions representing 1.8% of the total. It should be noted that a greater number of common analytes were observed in the intracellular compartment between IW and IM (609, 24.9%), compared to the IM and E fractions, which shared only 194 analytes (7.9%), and the IW and E fractions, which shared only 141 ions (5.8%).

Further fragmentation experiments were performed on these three cellular fractions using LC-MS/MS positive and negative ionization modes. Out of a total of 2441 analytes detected by simple LC-MS experiments, 1530 could have been analyzed by LC-MS/MS. These MS/MS fragmentation data were used for metabolite annotation (Table S2) and molecular network construction (Figure 2d). In this network, each analyte is represented by a node in which the diameter corresponds to its respective total ion intensity, while the color indicates the portion of the cellular fraction in which it has been detected. Clouds group ions together that present similar MS/MS fragmentation profiles according to the GNPS/MetGem algorithm. The most important clusters were comprised of molecules isolated from the supernatant possessing COOH acid groups such as cepteic, roccellic, and glutamic acids. Remarkably, the E fraction also contained various fatty acids (roccellic acid) and amino derivatives of fatty acids (erucamide). On the intracellular side, the metabolites that were isolated with water (polar, in blue) included hydrophilic compounds with osidic residues such as nucleosides, malto-pentose, and glycan tri-saccharides, while those extracted with methanol (non-polar, in green) comprised hydrophobic molecules, mainly lipids such as glycerolipids or erythrosphingosines. Other components representing intermediary hydrophobicity that were retrieved in both the IM and IW fractions, but remarkably not in the supernatant, included the dolastatins, microginins, and various amino acids or peptides, among others.

### 2.2. Dynamics of Metabolite Production under Normal (Control), Higher Light, and Higher Temperature Conditions

#### 2.2.1. Influence of Growth on Metabolite Dynamics

**Figure 3:**
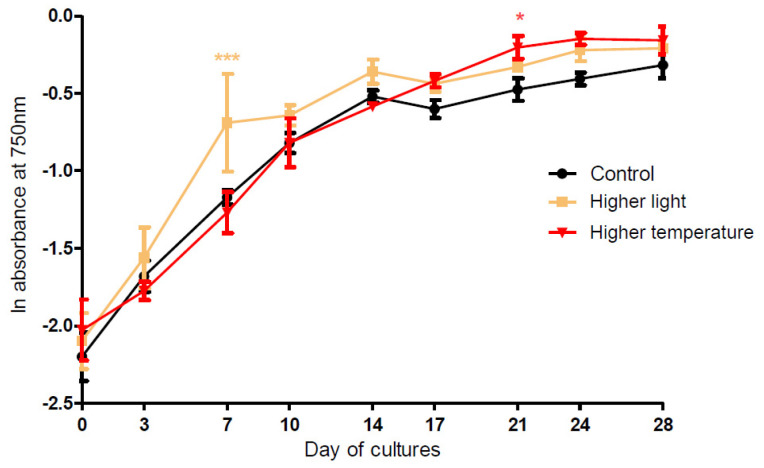
Cell growth curves of *Aliinostoc* sp. PMC 882.14 according to different culture conditions (“control” 24 °C/12 µmol·m^−2^·s^−1^, “higher light” 24 µmol·m^−2^·s^−1^, “higher temperature” 28 °C) according to the number of culture days considering the in vivo absorbance at 750 nm (mean ± SD). Significant differences between control and treatments (“higher light” and “higher temperature”) were assessed by the ANOVA (*p* < 0.001 for higher light at D7 and *p* < 0.05 for the higher temperature at D21) corrected with Bonferroni’s test.

During the 28 days of PMC 882.14 *Aliinostoc* sp. culture, the growth as measured by A750 nm (Figure 3), chlorophyll-a concentration, and cell counts (Figure S2) in the three experimental conditions showed similar patterns with an exponential phase from D0 to D14, followed by a plateau into stationary phase after D14. Comparison of the experimental growth curves with those obtained under control conditions (24 °C, 12 µmol·m^−2^·s^−1^ light) showed some significant variations with greater growth of *Aliinostoc* exposed to higher light intensity (24 µmol·m^−2^·s^−1^) at D7 (exponential phase, *p* < 0.001) and to higher temperature (28 °C) at D21 (stationary phase, *p* < 0.05).

**Figure 4:**
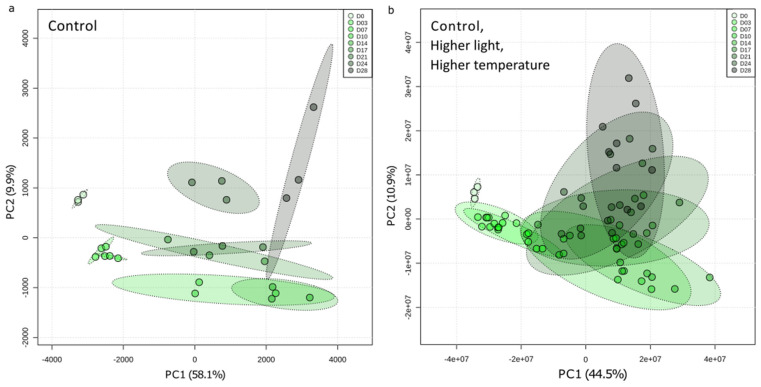
Principal component analysis (PCA) representing the evolution of the intracellular metabolic composition of *Aliinostoc* sp. PMC 882.14 as a function of the number of culture days (**a**) under the control condition only, or (**b**) under control, “higher light”, and “higher temperature” conditions. Each point represents a culture replicate. The green gradient reflects the temporal scale of the cultures.

Regarding the intracellular metabolome (pooled IM and IW fractions), the variations in global metabolome composition during the 28 days of culture were first explored with unsupervised multivariate PCA for control condition only (Figure 4a), and then for all three experimental conditions (Figure 4b). A progressive shift in the global metabolome during the duration of the culture was observed. Although the closeness of the measurements confirms reliable replication of the experiments, it also reveals an increasing disparity between triplicates through the growing. However, the points corresponding to the first days of sampling (D0, D3, and D7) are very similar, suggesting that only small changes in metabolic composition occur during the first seven days of culture. Interestingly, the PCA analyses mostly discriminated the samples from D0 to D14 according to Component 1 (representing 58% and 45% of the total variance, respectively), while Component 2 discriminated samples from D14 to D28 (10% and 11% of the total variance, respectively). This suggests that two metabolome regulation events (Metabolic Phase 1, from D0 to D14, and Metabolic Phase 2, from D14 to D28) could be distinguished over the 28-day period.

Similarly, supervised multivariate analysis (partial least squares–discriminant analysis, PLS–DA) was then conducted with respect to the days of sampling first for control samples only (Figure S4a) and then for samples from the three experimental conditions (Figure S4b). The individual PLS–DA plots exhibit patterns very similar to those observed for PCA (also with similar relative percentage contributions of Components 1–2), confirming good performance (R^2^_cumulative_) and predictability (Q^2^_cumulative_) scores. Figure S4c,d provides a list of the analytes that are contributing the most to sample discrimination during the culture period (variable of importance in the projection, VIP score > 2). Interestingly, the variables that most discriminate samples during growth kinetics are globally the same when considering the samples from the control condition only, or those from the three experimental conditions (bordered by red squares). Except for some rare examples, these intracellular metabolites display a global net increase in relative concentration, suggesting that they accumulate within cells during culture (Figures S4–S7). Analysis of individual analyte variations illustrates this global tendency to increase over time of culture and allows discrimination into three principal patterns: (1) one-step progressive increase at D10, as observed for some microginins and dolastatins, and for adenosine (Figure S5a); (2) a two-step progressive increase at D3 and D17, as observed for microviridin 1724 (Figure S5b,c); and a late, but significant increase at D24, such as for the monoacylglycerol 16:1 (Figure S5c). These observations suggest that various regulatory processes controlling the intracellular content of those components may occur during culture, depending on the molecular family and the balance between its biosynthesis and consumption within the cyanobacterium.

#### 2.2.2. Influence of Light and Temperature Variations on the Metabolomic Dynamics

**Figure 5:**
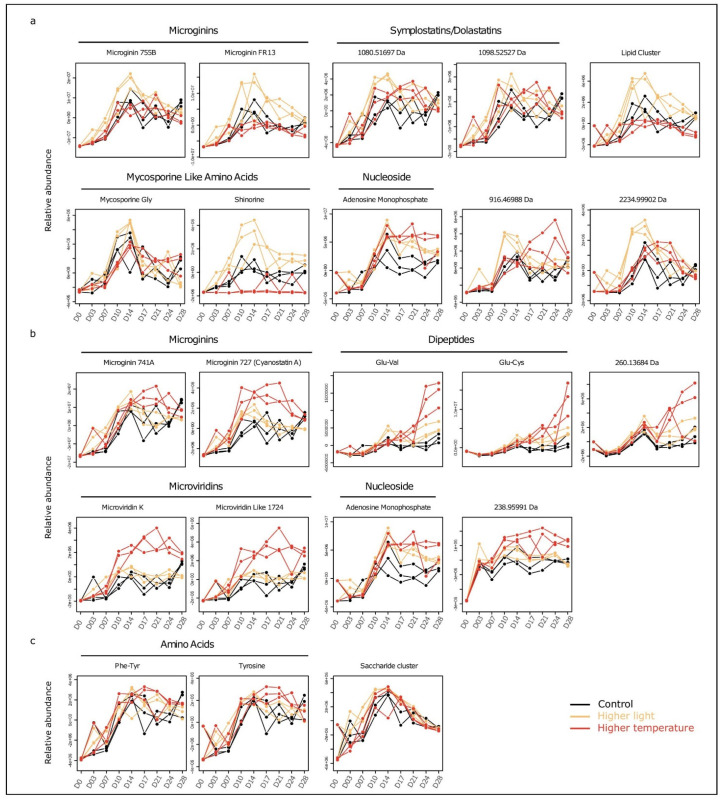
Relative abundance profiles of a selection of 22 analytes among the 48 presenting the best MEBA (multivariate empirical Bayes analysis) classification scores, further confirmed by two ways ANOVA (*p* < 0.001). (**a**) Metabolites with a higher intracellular concentration in the “higher light” condition than in the control. (**b**) Metabolites with a higher intracellular concentration in the “higher temperature” condition than in the control. (**c**) Metabolites presenting a more complex pattern of regulation when compared to the control. Each line representing a different replicated culture.

PCA analysis revealed that metabolite composition was controlled by culture conditions (Figure S3). To identify the metabolites whose relative levels were the most modified by light and temperature, data were analyzed by a multivariate approach specific for time series investigation (MEBA, multivariate empirical Bayes analysis). This approach pinpoints those variables showing the largest variation in level over time between the different experimental conditions. Results were supported by two-way ANOVA (*p* < 0.001), which revealed a crosswise effect of experimental conditions and culture duration. The variation profiles of the metabolites presenting the best MEBA scores are illustrated in Figure 5 and Table S3. Figure 5a depicts those metabolites whose relative intracellular concentration was greater under higher light conditions compared to control. Overall, these metabolites (two microginins and two dolastatins) follow a similar trend, characterized by an increase until D14, then a slight decrease. For these components, the peak of maximal concentration at D14 was also observed under control conditions but was less marked. Interestingly, one of the metabolites belonging to the MAA family, shinorine, a potential photo-protector, exhibited a greater intracellular accumulation than control under higher light intensity, while almost no signal was detectable under higher temperature. Figure 5b shows the metabolites whose intracellular content was greater under higher temperature compared to control. The two microginins, two microviridins, adenosine monophosphate and an unknown metabolite of 238.95991 Da (neutral mass), showed an initial relative concentration increase up to D10, followed by a plateau through D28. Markedly different profiles were observed for the dipeptides, Glu-Val and Glu-Cys, as well as for an unknown 260.13684 Da metabolite that showed a significant concentration increase after D21. The maximum increase of intracellular metabolite concentration seemed to be induced under higher light conditions by 10–14 days of culture, after which the concentration decreased. In contrast, temperature-induced increases occurred later in the period after D14 but appeared to be much more stable until D28.

On the extracellular side, similar analyses were attempted for the extracellular analytes (Figures S8–S10) and lead to the following observations: (i) the extracellular metabolome presented a net temporal variation, with important heterogeneity between replicates at stationary phase (Figure S8); (ii) the experimental variables higher light and higher temperature seemed to have a limited effect on variation of the extracellular metabolome (Figure S9); (iii) the analytes showing the best discrimination with respect to sampling time were the same when considering only the control condition or all conditions together (Figure S10); (iv) the extracellular components included mostly unknown molecules presenting global increases or decreases during the growth period (Figure S11); and (v) no perceptible metabolite transfer from the intracellular compartment to the extracellular one was observed over the investigated time period that covered both exponential and stationary growth phases.

## 3. Discussion

Analysis of the molecular network of metabolites of *Aliinostoc* sp. PMC 882.14 indicated the presence of numerous common cellular metabolites such as dipeptides, nucleosides, and fatty acids but also molecules specific to cyanobacteria such as analogues of MAAs, somamides, microviridins, and microginins. Somamides are members of the class of cyclo-depsipeptides and have been isolated in particular from cyanobacteria of the genus *Schizotrix* and *Lyngbya* [22]; however, this family of molecules has been poorly described so far. *Aliinostoc* sp. PMC 882.14 also produces different variants of microginins (Figure S1). These molecules are secondary metabolites of linear peptide structure synthesized via the NRPS/PKS hybrid biosynthetic pathway [23], which can lead to the formation of a large array of structural variants by a single strain [24]. To date, more than 90 variants of microginins have been referenced in databases [25], isolated mainly from cyanobacteria of the genus *Microcystis* and *Planktothrix* but also some cyanobacteria belonging to the genus *Nostoc* [26].

Measuring the metabolome variations over a culture period of 28 days under different experimental conditions revealed that time of culture was the main driver controlling the relative composition of both intra- and extra-cellular contents of *Aliinostoc* sp. PMC 882.14. The samples corresponding to the different points of the time series were discriminated along with Component 1 for samples ranging from D0 to D14, then along with Component 2 for later samples (Figure 4). This progressive temporal change of the intracellular metabolite contents across two distinguishable phases (D0–D14 and D15–D28) was in perfect correspondence with the growth phases (exponential phase from D0 to D14, then stationary phase from D15 to D28) observed from monitoring growth of the cultures (Figure 3 and Figure S2). A closer look at the evolution, through the time series, of the concentrations of the variables responsible for the global metabolome variations (Figures S4 and S5) reveals a global increase in their relative intracellular concentration. It also highlights the existence of even more subtle regulation processes probably involving biosynthesis, accumulation, and consumption events (Figure S5a–c). Interestingly, most metabolites belonging to the same molecular family evinced very similar variation patterns, suggesting the presence of homogeneous regulatory processes affecting all the different variants of each molecular family. While certain analytes, such as microginins, exhibited a prompt and important increase in their relative concentration at the end of the exponential phase followed by stabilization, the relative concentration of other molecules such as microviridins increased by formation of a transitional step between D10 and D21. In contrast, certain metabolites presented a mainly net increase during the late stationary phase (D24–D28).

Quorum sensing (QS) is largely considered to be involved in the regulation of metabolite synthesis by microorganisms [27], and we assume that this process might be moderately involved in the variation of the metabolites observed in the present work. Indeed, it appears that only very limited differences in cellular concentrations could have been observed between the various cultures performed under different light and temperature conditions (Figure 3 and Figure S2), supporting the idea that the metabolite content variations were induced by the differences in light and temperature conditions.

The physical stimuli induced by higher light (24 µmol·m^−2^·s^−1^) or higher temperature (28 °C) seem to have different cellular impacts since the accumulation of intracellular metabolites differs in quantity and kinetics between both conditions (Figure 5). In the present case, the higher light level induced more rapid metabolite variation than the higher temperature.

The maximum induction of microginin 755B and microginin FR13, symplostatin/dolastatin, MAAs (shinorine and mycosporine-glycine), as well as certain non-annotated metabolites under higher light conditions (24 µmol·m^−2^·s^−1^) occurred between D10 and D14, corresponding to the end of the exponential phase (Figure 5a). One can here hypothesize that the light induction of these components could be promoted by the enhanced photosynthesis that occurs under high light conditions. Interestingly, shinorine and mycosporine–glycine has been already reported for antioxidant activity, and this metabolite could play a role in reducing the oxidative stress effects potentially induced by the higher light level [28]. We can then propose that the light induction of such antioxidant metabolites could serve at protecting the cells from light-induced oxidative stress and might be degraded through this defensive process. Furthermore, an addition experimental condition performed under extreme high light condition (48 µmol·m^−2^·s^−1^, data not shown) led to the rapid senescence of the *Aliinostoc* culture (within less than 10 days), supporting the idea that a cellular oxidative stress may already occur under “higher light conditions” (24 µmol·m^−2^·s^−1^). Under the higher temperature growth condition (28 °C instead of 24 °C), however, other compounds present net intracellular concentration increases during the early (from D14) or the late (from D24) parts of the stationary phase, as exemplified by microginin 741A, microginin/cyanostatin A, microviridin K, microviridin 1724, and two dipeptides (Glu-Val and Glu-Cys) (Figure 5b). In contrast to the light-induced metabolites, the concentrations of the temperature-induced metabolites appear to be less important and to increase only at the end of the exponential phase, or even later. The difference of only 4 °C between the control (24 °C) and the higher temperature conditions (28 °C) appears to have a less general impact on the metabolite production and the growth of *Aliinostoc* sp. PMC 882.14 than the increase in light from 12 to 24 µmol·m^−2^·s^−1^. The opposite result was described in 2011 by Kumar et al. with regard to pigment production in *Spirulina platensis* [29]. The large increase in concentration of the two MAAs, shinorine and mycosporine–glycine, under increased light intensity was consistent with a putative photo-protective role of MAAs against UV-B radiation [30]. However, MAAs may have multiple physiological functions in cyanobacteria as they may act more generally as antioxidants or osmo-protectors [31].

The relative variations in the intracellular metabolite contents could result from a combination of different cellular processes [32,33]. We can assume that metabolites undergo the sum of biosynthesis, degradation, and secretion. Most of the latter supposition appears unlikely in the present case as analysis showed no apparent metabolite transfer from the intra- to the extra-cellular compartment. However, the characterization of the peculiar cellular processes that affect the biosynthesis and the degradation of metabolites remains largely un-explored for these micro-organisms and would require further specific investigations. In addition, a few metabolites presented a late decrease (D14–D28) in their respective intracellular contents (Figure 5c), which did not seem to be related to secretion. We hypothesized that these metabolites might have been degraded during the late growth phase, thus resulting in lower substrate availability in the growth medium during the experiment. Adenosine monophosphate (AMP) is a primary metabolite that was present at higher relative concentrations during the stationary phase under the tested light and temperature conditions compared to the control (Figure 5). This accumulation could be the result of the degradation of ADP (adenosine diphosphate) and ATP (adenosine triphosphate), which may indicate an increase in energy consumption occurring under stress [34]. However, to confirm this hypothesis, it would be necessary to rigorously quantify ADP and ATP. The exact quantities of these compounds were not retrieved by the present non-targeted metabolomics pipeline, which was simply based on variable extraction and differential analysis to calculate the ratios between the amounts of AMP, ADP, and ATP. Even if most metabolites belonging to the same family exhibit the same dynamics, some peptide variants seem to be produced preferentially to others depending on culture conditions, as is the case for some microginin variants. Differences in the ratios of variants of secondary metabolites in other cyanobacteria have already been described, especially in the case of response to light stress [28]. In their 2005 study of *Planktothrix agardhii*, Tonk and co-workers observed a light-stress-induced change in the ratios of different variants of microcystins, cyanobacterial-specific secondary metabolites produced by NRPS/PKS complexes, similar to those responsible for microginin biosynthesis. Variations in the intracellular availability of the substrates necessary for the biosynthesis of these compounds could be an explanation for such differences. Indeed, microginins 755B and 741 and microginin/cyanostatin A differ in the identity of the central amino acid (alanine, serine, or valine, respectively), and which variant is synthesized varies depending on the affinity of the elongation enzymes for the substrates and their respective availability [18].

## 4. Materials and Methods

### 4.1. Biological Material

*Aliinostoc* sp. PMC 882.14 strain was isolated in 2014 from the thermal mud of Balaruc-les-Bains (France) and kept in a collection at the Paris Museum Collection (PMC, Paris. Four large volumes (500 mL) of pre-cultures were cultivated in 1 L Durand’s bottles for up to 46 days in Z8 culture medium, with 12 µmol.m^−2^.s^−1^ light intensity and 12 h:12 h light-dark photoperiod. Samples were collected after 33 days for chemical strain characterization. Pre-cultures bottles were thus pooled (on the 46th day) to obtain a large volume (above 2 L) of homogeneous stock solution (D0) that was further used for the experimentation. From this pool, the 1/10 diluted cultures were then inoculated in triplicates in 1 L bottles (initial volume of 500 mL) for experimentations under “control”, “higher light”, and “higher temperature” conditions during the 28 following days. Figure 1 represents the global experimental and analytical pipeline developed in this study.

### 4.2. Culture Conditions and Sampling

Experiment was conducted over a 28 day period in “control” (24 °C, 12 µmol·m^−2^·s^−1^), “higher light” (24 °C, 24 µmol·m^−2^·s^−1^), and “higher temperature” (28 °C, 12 µmol·m^−2^·s^−1^) conditions. Experimental conditions were performed in triplicate, under a 12 h:12 h photoperiod and a constant homogenization maintained by magnetic agitators. Two weekly samples were taken throughout the 28 days (D0, D3, D7, D10, D14, D17, D21, D24, and D28). Each sampling (22 mL) was subdivided into several parts for (i) the extraction and the analysis of intra- and extra-cellular metabolites (10 mL), (ii) the biomass monitoring using extraction and quantification of chlorophyll pigments (10 mL), and optical density and cell counts (2 mL). A sampling at D0 was performed on the diluted (1/10) stock solution, in triplicate.

### 4.3. Biomass Dynamics

Cyanobacterial biomass was followed using absorbance at 750 nm by in vivo spectrophotometric (Agilent Cary 500; Santa Clara, CA, USA), chlorophyll-*a* concentration [35] and cell abundance by Malassez’ cell counts [36]. Data have been transformed into a Neperian logarithm, and statistical analysis between treatments and the control (ANOVA with Bonferonni’s correction) was conducted with GraphPad Prism 5.01 software.

### 4.4. Metabolite Extractions

Samples (10 mL) were centrifuged (10 min, 3220× *g*, 4 °C). The supernatant was isolated and stored at −20 °C in the prevision of extracellular metabolites liquid extractions. The pellet was centrifuged and stored at −80 °C for at least 24 h, and then the sample lyophilization was performed (−50 °C, 0.02 mBar for 16 h) (Labconco, Kansas City, MO, USA). Dry weight was thus determined, and then the two sequential extractions were performed (with 100 µL of solvent for 1 mg biomass dry weight). The first extraction of hydrophilic metabolites was carried out with a water/formic acid mixture (0.1%), and a second extraction targeting hydrophobic metabolites was performed with a water/methanol mixture (75%); then, the two supernatants were pooled (apart from the pre-culture to compare metabolites composition in the three extraction fractions). For both extractions, cell lysis was performed by sonication: 3 cycles of 30 s, 10 s break between each cycle, at 80% of the maximum intensity (SONICS Vibra Cell, Newton, CT, USA; 130 Watt, 20 Khz). Samples were then centrifuged (10 min, 13,400× *g*, 4 °C), and the supernatants were collected and stored in obscurity at −20 °C before mass spectrometry analysis.

Extracellular metabolites present in the supernatant of the culture media (D0, D7, D14, D21, and D28 as they correspond to key moments of the growth phases) were extracted by liquid/liquid extraction with the equivalent volume of solvent (100% ethyl acetate) with a separating funnel. The upper organic phase has been collected after homogenization and degassing. Water molecules in the organic phase were removed by the gradual addition of magnesium sulfate powder (MgSO_4_). Consecutively to filtration, the liquid was dried under airflow, and then the dry extracts were re-suspended in a mixture of water/methanol (75%)/formic acid (0.1%) and centrifuged (10 min, 13,400 *g*, 4 °C) before storage at −20 °C.

### 4.5. Mass Spectrometry Analysis

Extracts were separated using ultra-high performance liquid chromatography (UHPLC). For each sample, 2 μL was injected, and molecule separation was performed by a Polar Advance II 2.5 pore C18 (Thermo Fisher Scientific, Waltham, MA, USA) chromatographic column at a flow rate of 300 μL.min^−1^ under a linear gradient of acetonitrile acidified with 0.1% formic acid (from 5 to 90% in 15 min). Metabolites contents were analyzed using an electrospray ionization hybrid quadrupole time-of-flight (ESI-QqTOF) high-resolution mass spectrometer (Compact, Brucker, Bremen, Germany) in the range 50–1500 *m/z*.

### 4.6. Simple MS Mode Analyses

Compounds were initially analyzed in simple MS positive and negative modes, without quadrupole fragmentation. This mode of analysis notably allows observing a signal intensity of each ion (measured in number of detected counts over the time range necessary for its elution) that is directly proportional to the quantity of ions present in the different samples. This analysis was carried out in order to compare metabolites composition in the three extraction fractions on the pre-culture (representing a total 2441 intracellular analytes extracted with acidified water or methanol and extracellular analytes, when data were merged) and in the samples collected from the experimentation (for either extra- or pooled intra-cellular fractions, analyzed on positive mode only). MS data were processed using MetaboScape 4.0 software (Bruker, Bremen, Germany) for recalibration of each sample analysis (according to internal standard), peak detection and selection of ions whose intensity was greater than 5000 counts in at least 10% of the set of samples, and peak realignment. Furthermore, different states of charge and adducts were grouped together and the “area-under-the-peak” was determined in order to generate a unique global data matrix containing semi-quantification results for each metabolite in all analyzed samples’ peak for each analyte (characterized by the respective mean mass of its neutral form and its corresponding retention time).

### 4.7. MS/MS Mode Analyses

For qualitative investigation of metabolites, additional ion selection with the quadrupole and fragmentation was carried out in tandem by collision ion dissociation (CID) according to the autoMS/MS analysis, performed in positive mode. The ions with the highest intensities in single MS (MS1) were then selected by the quadrupole and fragmented in a collision cell. The resulting ions of the fragmentation of their respective parent were transferred and detected. This series of MS/MS datasets containing a fragment list of all selected ions allow qualitatively the characterization of the diversity of the corresponding metabolites produced by *Aliinostoc* sp. PMC 882.14. through the comparison of the fragmentation profiles. The files containing the fragmentation information for each ion analyzed were exported in mgf format using MetaboScape 4.0 (Bruker, Bremen, Germany) software before being used for the generation of the molecular network of structural similarity.

Metabolites annotations were made from MS/MS data (mgf file) by generating a molecular network for the comparison of fragmentation profiles using the MetGem software (version 1.1.2) and the GNPS algorithm [37]. Public and generalist spectral databases GNPS library, NIH Clinical collections, and EMBL metabolomics were used. Annotations were supplemented by a match with the CyanoMetDB database [25,38], which contains all the raw formulae of the 2100 cyanobacterial metabolites already described [25].

### 4.8. Statistical Treatments

The MetaboAnalyst 4.0 platform [39] was used to perform data matrix normalization (Pareto), principal component analysis (PCA), Partial least square discriminant analysis (PLS-DA), multivariate empirical Bayes analysis (MEBA) [40] and ANOVA (analysis of variance).

## 5. Conclusions

In a conclusion, this study provided the first picture of the diversity and dynamics of metabolites produced by the cyanobacterium *Aliinostoc* sp. PMC 882.14 under different culture conditions. This strain was characterized by the presence of numerous BGCs in its genome and the consecutive production of numerous and specific metabolites such as various analogues of somamides/dolastatins, microginins, microviridins, and MAAs. The growth phases related to the physiological status of the cells appear to play a key role in the metabolic composition and abundance, with the most statistical variations occurring between the exponential vs. stationary growth phases. Light and temperature also appear to have significant effects on the dynamic of the metabolic composition and should be further considered, especially in the context of a global metabolomic comparison between cyanobacteria and bioactive compound production. Indeed, for valorization purposes the production of potentially bioactive components, such as shinorine, somamides/dolastatins, and microginins, by *Aliinostoc* sp. PMC 882.14, represents great potential for further bioactivity screening taking into account cultural conditions.

## Data Availability

The datasets used and analyzed during the current study are available online from MassIVE database: ftp://massive.ucsd.edu/MSV000088276/.

## Supplementary Materials

The following are available online at https://www.mdpi.com/article/10.3390/metabo11110745/s1, Table S1: Biosynthetic gene clusters identified from *Aliinostoc* sp. PMC 882.14, Table S2: Metabolite annotations from MS/MS fragmentation data, Table S3: List of metabolites presenting the best MEBA scores (MB.statistics) with corresponding ANOVA analysis results. Variations over the time course for metabolites highlighted in yellow are represented in Figure S5 MW = Molecular Weight, RT = Retention Time. Figure S1: Candidate microginin biosynthetic gene cluster from *Aliinostoc* sp. PMC 882.14. Figure S2: Growth curves based on extracted chlorophyll *a* and cell count. Significant differences between control and conditions are represented by a single star (ANOVA, *p*.value < 0,05) or two stars (ANOVA, *p* value < 0.01). Figure S3: Principal Component Analysis (PCA) representing the evolution of the intracellular metabolic composition of *Aliinostoc* sp. PMC 882.14 as a function of culture conditions (control = grey, “higher light” = yellow and “higher temperature” = red) (a) PC1 and PC2 and (b) PC1 and PC3. Figure S4: PLS-DA considering the days of sampling for (a) control samples only and (b) samples from the three experimental conditions and (c,d) corresponding lists of the analytes contributing the most to the sample discrimination through the culture kinetics (variable of importance in the projection, VIP score > 2). The red-framed lines correspond to the metabolites in common with the analysis performed only on controls. Figure S5: Box-plots representing the 29 intracellular molecules (exhibiting VIP scores > 2; Figure S4d), which explain most of the differences between the different days of culture considering samples from the three experimental conditions. Figure S6: Box-plots representing the 38 intracellular molecules (exhibiting VIP scores > 2; Figure S4c), which explain most of the differences between the different days of culture considering only control samples. Figure S7: Principal Component Analysis (PCA) representing the evolution of the extracellular metabolic composition of *Aliinostoc* sp. PMC 882.14 as a function of the number of culture days (a) under the control condition, (b) under control, “higher light” and “higher temperature” conditions. Each point represents a culture replicate. The green colour gradient reflects the temporal scale of the cultures. Figure S8: Principal Component Analysis (PCA) representing the evolution of the extracellular metabolic composition of *Aliinostoc* sp. PMC 882.14 as a function of culture conditions (control = grey, “higher light” = yellow and “higher temperature” = red) (a) PC1 and PC2 and (b) PC1 and PC3. Figure S9: PLS-DA representing the evolution of the extracellular metabolic composition of *Aliinostoc* sp. PMC 882.14 considering the days of sampling for (a) control samples only and (b) samples from the three experimental conditions and (c,d) corresponding lists of the analytes contributing the most to the sample discrimination through the culture kinetics (variable of importance in the projection, VIP score > 2). The red-framed lines correspond to the metabolites in common with the analysis performed only on controls. Figure S10: Box-plots representing the 25 intracellular molecules (exhibiting VIP scores > 2; Figure S10d), which explain most of the differences between the different days of culture considering samples from the three experimental conditions.
