# Supplementary material for: Dynamics of the Metabolome of Aliinostoc sp. PMC 882.14 in Response to Light and Temperature Variations
Source: Metabolites. 2021 Oct 29;11(11):745. doi: 10.3390/metabo11110745 (PMC8618397; doi:10.3390/metabo11110745)

Table S1 **Biosynthetic gene clusters identified from *Aliinostoc* sp. PMC 882.14.**

Cluster	Contig	From	To	Taille (bp)	Cluster type	Most similar known cluster (antiSMASH prediction)	Similarity	BGC expert annotation
1	C882_NODE_3 - Region 1	15 640	37 498	21 858	NRPS-like	Shinorine	100%	**Shinorine**
2	C882_NODE_3 - Region 2	245 505	310 129	64 624	NRPS/T1PKS/microviridin	Puwainaphycins	60%	**Microginin**
3	C882_NODE_3 - Region 3	640 730	684 201	43 471	NRPS-like	X	X	Unknown
4	C882_NODE_3 - Region 4	1 403 211	1 493 957	90 746	NRPS/T1PKS	Hapalosin	40%	Peptide
5	C882_NODE_3 - Region 5	1 568 034	1 637 956	69 922	NRPS	Cyanopeptin	75%	Peptide
6	C882_NODE_7 - Region 1	82 074	126 088	44 014	T1PKS	Amycomicin	62%	Amycomicin-like
7	C882_NODE_7 - Region 2	166 759	210 645	43 886	NRPS	Micropeptin	62%	Unknown
8	C882_NODE_7 - Region 3	264 995	311 442	46 447	T1PKS/bacteriocin	Jamaicamide	15%	Unknown
9	C882_NODE_7 - Region 4	404 145	473 658	69 513	NRPS	Nostopeptolide	37%	Peptide
10	C882_NODE_7 - Region 5	560 196	581 026	20 830	Terpene	X	X	**Carotenoid**
11	C882_NODE_9 - Region 1	145 215	197 610	52 395	T1PKS/hglE-KS	Heterocyst glycolipids	85%	**Heterocyst glycolipids**
12	C882_NODE_9 - Region 2	335 457	345 690	10 233	Bacteriocin	X	X	Bacteriocin
13	C882_NODE_9 - Region 3	364 924	406 075	41 151	Ladderane	X	X	Ladderane
14	C882_NODE_12 - Region 1	482 095	502 947	20 852	Microviridin (bacteriocin)	Microviridin K	62%	**Microviridin**
15	C882_NODE_13 - Region 1	45 863	138 173	92 310	NRPS/T1PKS	Nostophycin	27%	Unknown
16	C882_NODE_14 - Region 1	225 206	244 913	19 707	Terpene	6,6'-oxybis(2,4-dibromophenol) / bartolosides	21%	Unknown
17	C882_NODE_14 - Region 2	305 160	326 092	20 932	Terpene	X	X	**Carotenoid**
18	C882_NODE_15 - Region 1	193 638	269 628	75 990	NRPS	Puwainaphycins	40%	Unknown
19	C882_NODE_15 - Region 2	270 364	315 220	44 856	T1PKS	Carbamidocyclophane	30%	Unknown
20	C882_NODE_16 - Region 1	202 309	253 885	51 576	NRPS/betalactone	X	X	Unknown
21	C882_NODE_18 - Region 1	65 575	76 344	10 769	Bacteriocin	Welwitindolinone	6%	Bacteriocin

Biosynthetic gene clusters (BGCs) have been identified in a previous study [21]. This table shows information from AntiSMASH about the BGCs identified in *Aliinostoc* sp. PMC 882.14. Expert annotation of the BGCs was performed and 6 BGCs were formally annotated (in green), 7 were partially annotated (orange) and 8 were unknown (red).

Table S2 **Metabolite annotations from MS/MS fragmentation data.**

Metabolite annotations	m/z parent	SHARED_NAME	FEATURE_ID	RT (s)
Tribenzylamin	226.12279	446.00	730.00	663
Gabapentin	226.17985	1181.00	1953.00	1017
L-cysteic acid	228.15998	857.00	1518.00	556
Linoleic acid	228.19584	634.00	1156.00	957
L-cysteic acid	242.1762	1236.00	2054.00	603
Acide Glutamique	244.18975	880.00	1546.00	606
Rimantadine	244.19047	1189.00	1963.00	1030
hydroxy-2-octylpentanedioic acid M	246.16974	816.00	1465.00	472
Glu-Cys	247.12837	80.00	211.00	125
Roccellic acid	256.15616	1254.00	2079.00	561
Glu-Cys	261.14378	776.00	1416.00	225
Glu-Cys	261.14393	777.00	1417.00	249
Roccellic acid	270.17153	838.00	1493.00	509
Lonidamine	272.95879	1276.00	2106.00	43
Triphenylphosphine	280.22665	1016.00	1724.00	824
Triphenylphosphine	280.26224	1043.00	1765.00	855
Triphenylphosphine	280.26298	1116.00	1865.00	927
Roccellic acid	282.17129	1280.00	2111.00	563
Erucamide	282.27789	1173.00	1941.00	1001
Triphenylphosphine	282.27824	558.00	946.00	841
Erucamide	282.27853	1161.00	1925.00	987
Octadecadien-1-ol	284.29421	688.00	1264.00	1028
D-erythro-C18-Sphingosine	297.24355	1296.00	2131.00	860
Erucamide	310.30831	1197.00	1971.00	1078
Monoerucin	310.30964	1190.00	1964.00	1034
Anhydrobrazilic Acid	317.14923	848.00	1506.00	527
Anhydrobrazilic Acid	317.14949	413.00	684.00	608
Phe-Tyr	359.15998	338.00	569.00	478
Miconazole	362.94136	1339.00	2186.00	48
dimethyl 2,4-bis(4-hydroxyphenyl)cyclobutane-1,3-dicarboxylate	373.18471	1347.00	2195.00	826
dimethyl 2,4-bis(4-hydroxyphenyl)cyclobutane-1,3-dicarboxylate [M-H]-	373.18548	1348.00	2196.00	1006
Eudesmin	402.3554	444.00	728.00	662
Sorbitane Monooleate	432.36778	668.00	1233.00	1007
Glycan Le-A Trisaccharide	479.18704	91.00	228.00	155
Glycan Le-A Trisaccharide	497.19705	75.00	200.00	116
tert-Octylphenol nonaglycol	532.382	533.00	888.00	799
Octadecatrienoic acid, 3-(hexopyranosyloxy)-2-hydroxypropyl ester	535.3113	1426.00	2293.00	774
Octadecatrienoic acid, 3-(hexopyranosyloxy)-2-hydroxypropyl ester	537.32793	1427.00	2294.00	835
Hexopyranosylhexopyranosyl)oxy](phenyl)acetonitrile	549.16738	1431.00	2299.00	61
Triphenylphosphine oxide	558.42033	432.00	710.00	634
tert-Octylphenol nonaglycol	576.40832	530.00	884.00	798
tert-Octylphenol nonaglycol	620.43502	987.00	1685.00	794
O-.beta.-Galactopyranosyl-D-mannopyranose	649.21879	61.00	174.00	99
O-.beta.-Galactopyranosyl-D-mannopyranose	649.21914	754.00	1368.00	63
octadecatrienoyl-sn-glycerol	697.36457	1461.00	2340.00	706
Maltopentaose	749.21153	43.00	139.00	86
6.alpha.-Methylprednisolone	749.38687	549.00	924.00	831
Maltopentaose	813.28711	759.00	1378.00	83
Maltopentaose	830.3133	42.00	136.00	83
Maltopentaose	835.26892	758.00	1377.00	83
Maltopentaose	893.30652	62.00	175.00	99
Maltopentaose	911.264	45.00	141.00	87
Somamide	935.48665	279.00	489.00	429
Symplostatin	1099.53298	885.00	1554.00	614
D-Glucopyranoside	1137.39084	56.00	162.00	98
D-Galactopyranosyl-(1->6)-beta-D-glucopyranosyl	1299.44302	60.00	172.00	99

Table S3 **List of metabolites presenting the best MEBA scores (MB.statistics) with corresponding ANOVA analysis results. Variations over time course for metabolites highlighted in yellow are represented in Figure6. MW = Molecular Weight, RT = Retention Time.**

Annotation	MW (Da)	RT (s)	p value (Annova condition)	Significant effect (Annova condition)	MB statistics	Meba Rank
Symplostatin/Dolastatin 1080	1080,51697	617,89	**9,75E-17**	*	**10,755**	1
	574,37337	483,15	**1,41E-23**	*	**8,767**	2
	591,23822	126,39	**2,34E-14**	*	**8,4507**	3
	383,27814	478,45	**2,06E-16**	*	**8,3909**	4
Phe-Tyr	358,15268	478,47	**6,12E-18**	*	**4,2673**	5
Symplostatin/Dolastatin 1098	1098,52527	617,9	**1,55E-14**	*	**4,1965**	6
Adenosine monophosphate	347,06289	108,5	**3,25E-17**	*	**2,974**	7
Tyr	181,07489	478,48	**6,93E-16**	*	**2,9718**	8
	916,46988	430	**3,29E-17**	*	**2,7954**	9
Microginin 755B	755,44607	483,11	**1,22E-20**	*	**2,7903**	10
Microviridin cluster	1724,73744	447	**7,14E-15**	*	**2,569**	11
Mycosporine gly	245,08273	67,05	**3,71E-15**	*	**2,1385**	12
Microginin 741A	741,43029	478,45	**2,85E-19**	*	**1,9134**	13
Microginin Cyanostatin A	727,41576	469,36	**1,85E-18**	*	**1,0855**	14
Microviridin K	1634,70176	417,91	**5,55E-19**	*	**0,54826**	15
	560,35775	478,46	**5,13E-24**	*	**0,23169**	16
	260,13694	233,62	**7,80E-16**	*	**-0,02492**	17
	231,92143	45,66	**1,32E-26**	*	**-0,19771**	18
	589,22293	136,33	**3,18E-08**	*	**-0,34041**	19
	397,29385	483,13	**7,06E-19**	*	**-0,47849**	20
Glu-Cys	246,12134	129,55	**1,40E-18**	*	**-0,8154**	21
Microginin FR13	769,46212	492,57	**9,84E-12**	*	**-1,1977**	22
Lipid-cluster	588,38921	492,62	**1,03E-11**	*	**-1,7025**	23
(2S)-1-O-Palmitoyl-3-O-?-D-galactopyranosylglycerol	492,32944	844,59	**2,50E-12**	*	**-1,7978**	24
	238,95991	45,36	**1,72E-22**	*	**-1,9585**	25
	496,19052	121,41	**1,19E-07**	*	**-2,2128**	26
	808,35856	483,18	**1,41E-23**	*	**-2,6115**	27
	210,10298	775,05	**0,0004669**	*	**-2,7509**	28
Microginin 725	725,43589	481,46	**5,59E-13**	*	**-3,5675**	29
	555,13068	108,69	**3,41E-17**	*	**-3,6947**	30
	2234,99902	617,93	**7,69E-16**	*	**-3,8586**	31
	575,72028	617,9	**4,40E-13**	*	**-3,9654**	32
	794,34383	478,53	**4,21E-16**	*	**-4,2866**	33
	272,94757	45,78	**6,16E-23**	*	**-4,317**	34
	260,13684	257,54	**6,38E-11**	*	**-4,3709**	35
	328,26104	781,76	**3,30E-15**	*	**-4,5952**	36
	779,37852	478,48	**4,06E-23**	*	**-4,8149**	37
	267,09664	108,67	**1,84E-05**	*	**-5,1143**	38
	330,27651	844,59	**3,32E-12**	*	**-5,1681**	39
	461,2138	57,8	**1,18E-05**	*	**-5,5018**	40
	352,26149	775,35	**3,25E-09**	*	**-6,301**	41
Microviridin cluster	1643,30459	454,44	**1,98E-09**	*	**-6,4183**	42
	793,39387	483,24	**1,84E-13**	*	**-7,2629**	43
	1128,81511	377,47	**3,50E-12**	*	**-7,4437**	44
Saccharide cluster	1136,47465	617,94	**2,15E-15**	*	**-7,4493**	45
	249,93189	45,75	**4,38E-27**	*	**-7,4845**	46
	592,3837	459,91	**9,71E-16**	*	**-7,839**	47
Shinorine	332,12179	78,99	**3,65E-10**	*	**-7,9429**	48

Figure S1 **Candidate microginin biosynthetic gene cluster from *Aliinostoc* sp. PMC 882.14.**

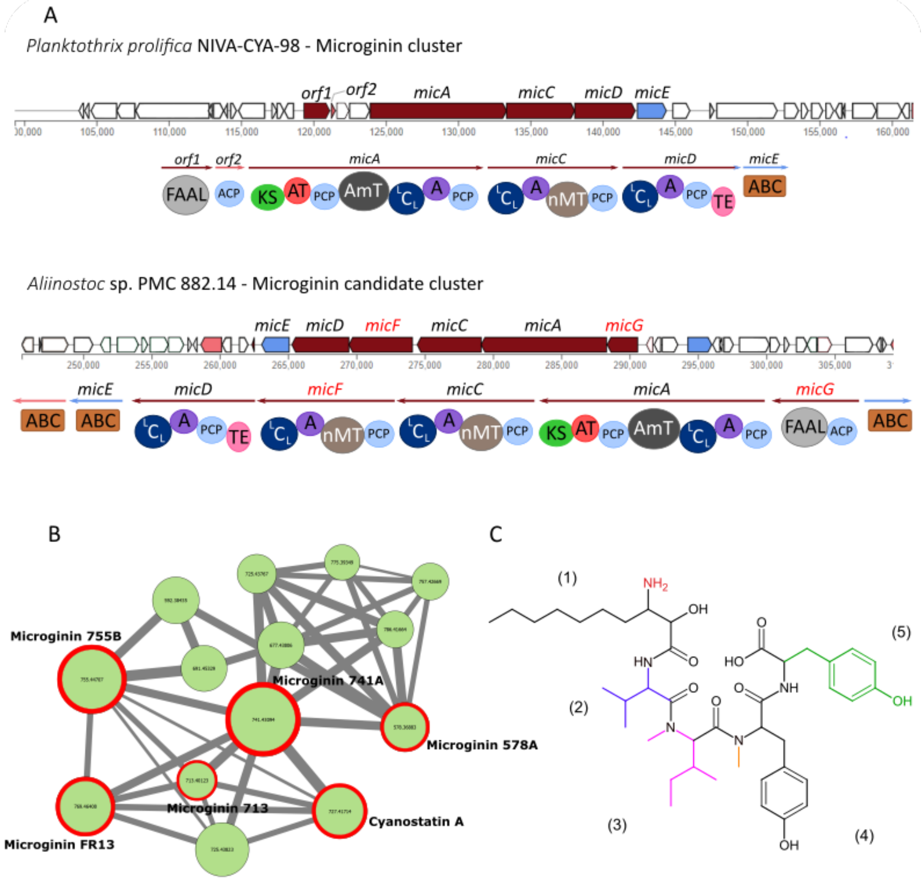

D

	MW	Amino acids sequence (1)	(2)	(3)	(4)	(5)
Peptide predicted	-	Pk-Mal	Val	MeIle	MeTyr	Tyr
Observed microginins						
Cyanostatin A	727.41714	Ahda	Ala	Val	MeTyr	Hty
Microginin 578A	578.36883	MeAhda	Ala	MeLeu/MeIle	Tyr	-
Microginin 713	713.40123	Ahda	Ala	Val	MeTyr	Tyr
Microginin 741A	741.43094	MeAhda	Ala	MeLeu/MeIle	Tyr	Tyr
Microginin 755B	755.44707	MeAhda	Ala	MeLeu/MeIle	MeTyr/Hty	Tyr
Microginin FR13	769.46408	MeAhda	Val	Leu/Ile	MeTyr	Tyr

A) Microginin biosynthetic gene cluster and NRPS/PKS synthetase organisation from *Planktothrix prolifica* NIVA-CYA-98 compared to candidate microginin BGCs from *Aliinostoc* sp. PMC 882.14.
B) Microginin cluster from molecular network (LC-MS/MS analysis and MetGem software) [21].
C) Predicted structure of the microginin variant potentially produced by *Aliinostoc* sp. PMC 882.14.
D) Detailled amino acids sequences of the microginins identified by our molecular network analysis compared with predicted structure of *Aliinostoc* microginin.

Figure S2 **Growth curves based on extracted chlorophyll a and cell count. Significant differences between control and conditions are represented by a single star (ANOVA, pvalue < 0,05) or two stars (ANOVA, *p*-value < 0,01).**

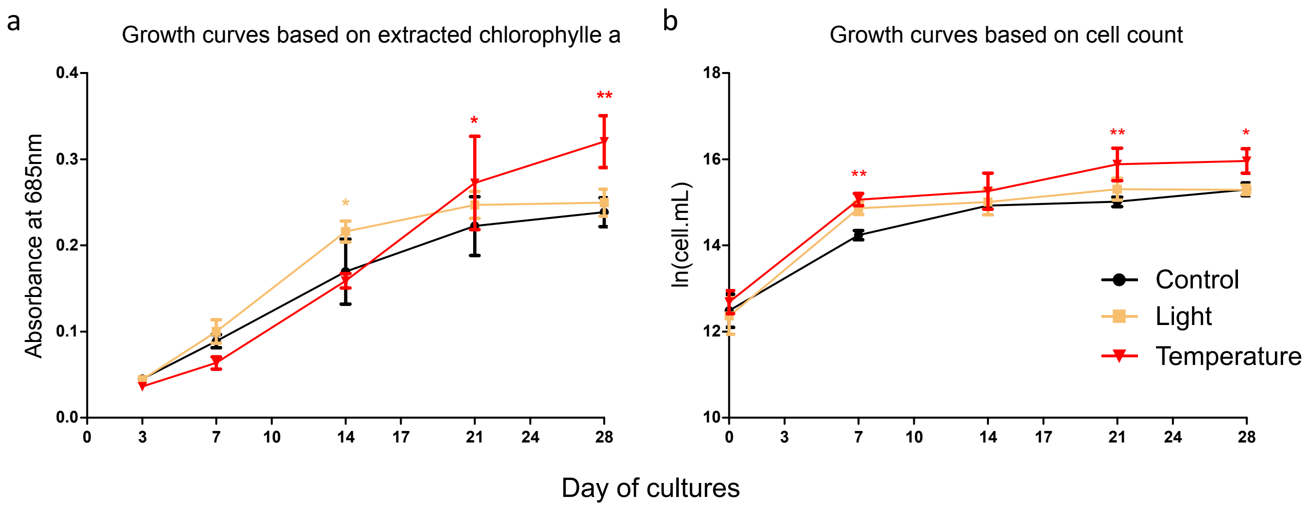

Figure S3

Principal Component Analysis (PCA) representing the evolution of the intracellular metabolic composition of *Aliinostoc* sp. PMC 882.14 as a function of culture conditions (control= grey, "higher light"= yellow and "higher temperature"= red) a) PC1 and PC2 and b) PC1 and PC3.

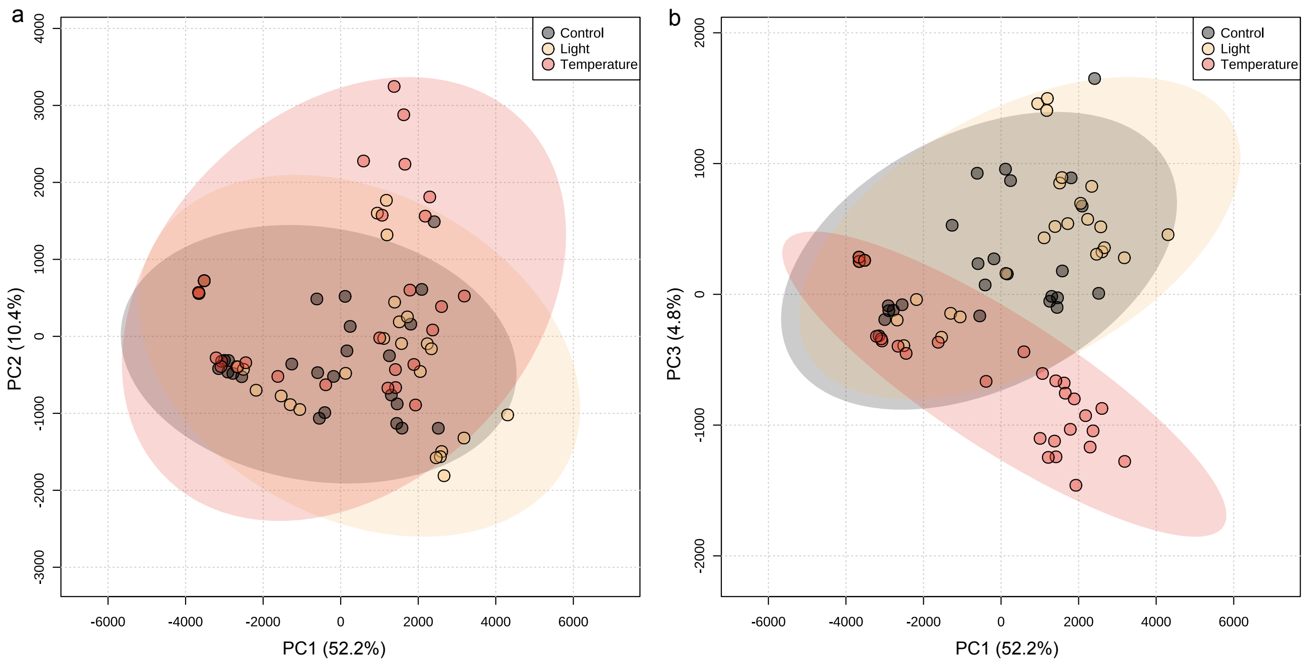

Figure S4 **PLS-DA considering the days of sampling for (a) control samples only and (b) samples from the three experimental conditions and (c,d) corresponding lists of the analytes contributing the most to the sample discrimination through the culture kinetics (variable of importance in the projection, VIP score >2). The red framed lines correspond to the metabolites in common with the analysis performed only on controls.**

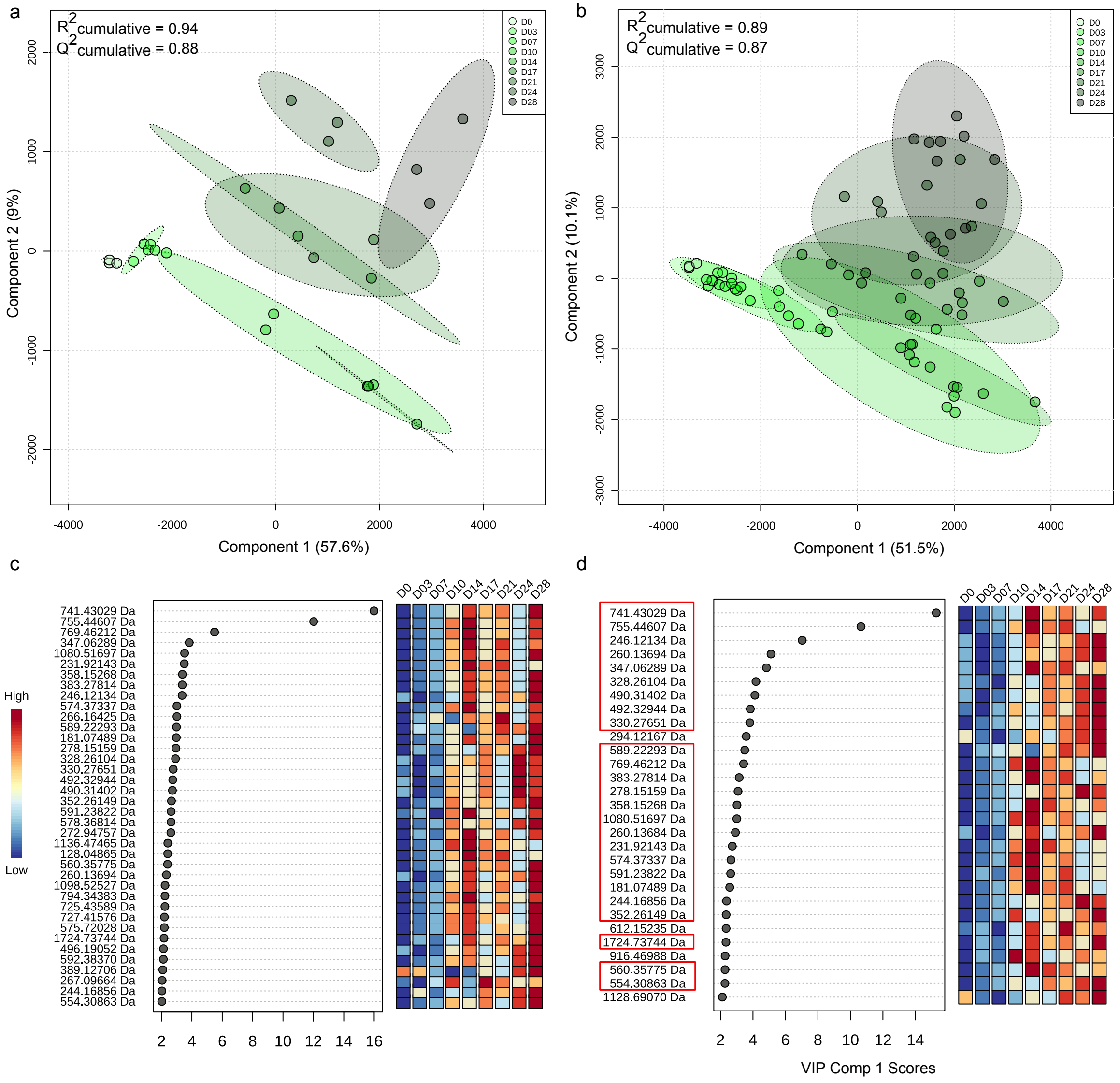

Figure S5 **Box-plots representing a selection of 15 among the 38 intracellular molecules (exhibiting VIP scores > 2), which explain most of the differences between the different days of culture. The three main patterns were illustrated with molecules whose relative intensity a) increases promptly at D14, b) increases by forming a transitional step between D10 and D21, c) increases mostly at the end of kinetics (D24 and D28).**

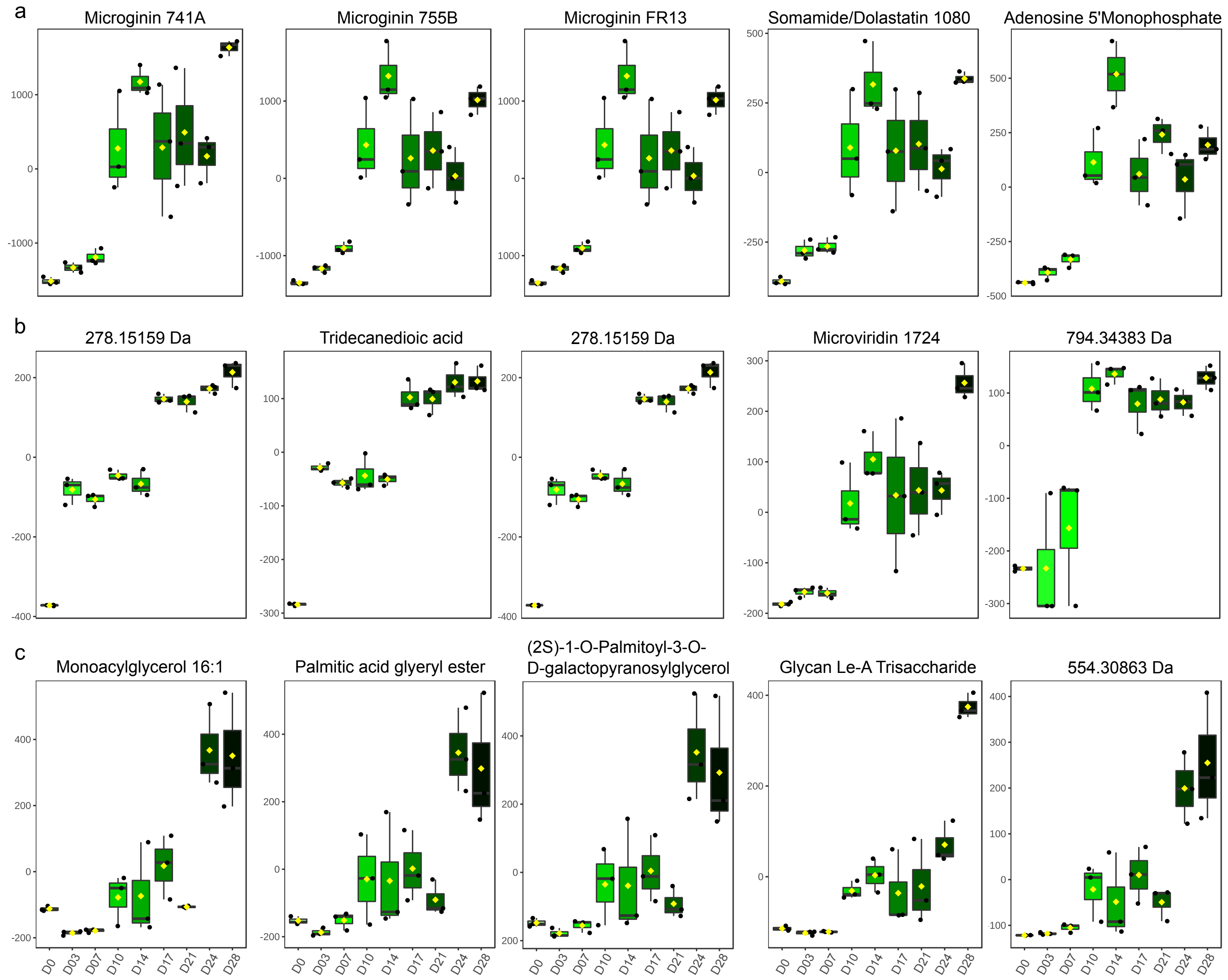

Figure S6

Box-plots representing the 29 intracellular molecules (exhibiting VIP scores > 2; Fig S4d), which explain the most of the differences between the different days of culture considering samples from the three experimental conditions.

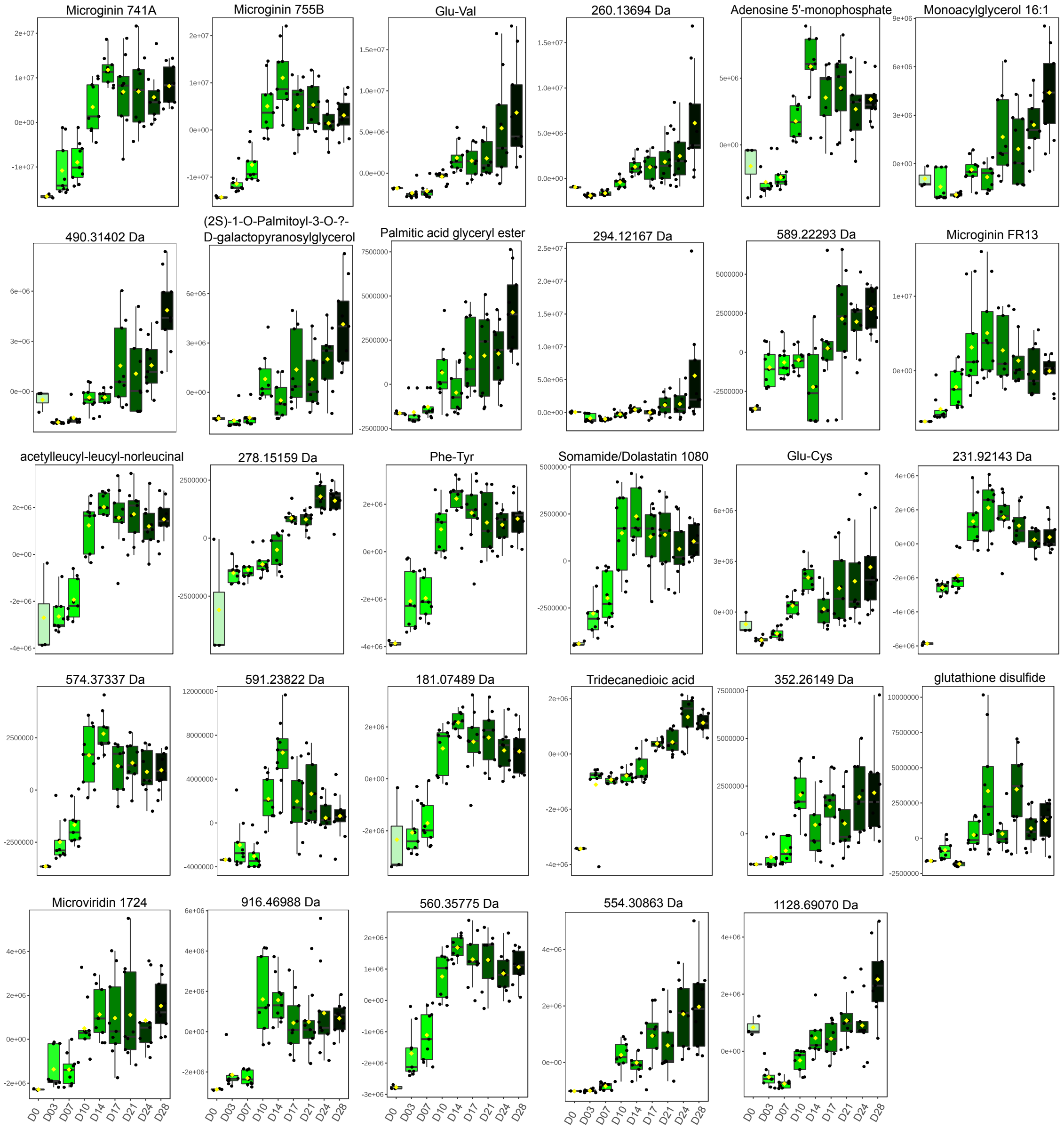

Figure S7

Box-plots representing the 38 intracellular molecules (exhibiting VIP scores > 2; Fig S4c), which explain the most of the differences between the different days of culture considering only control samples.

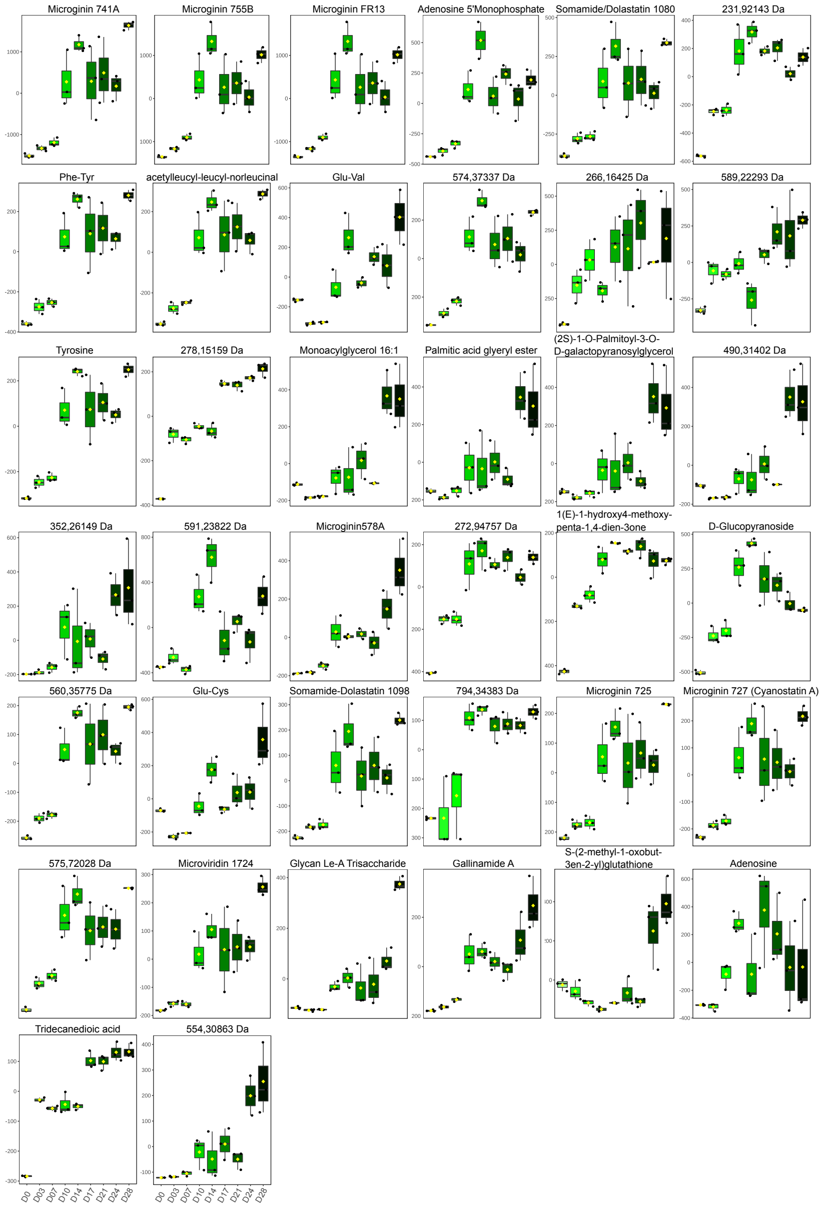

Figure S8 **Principal Component Analysis (PCA) representing the evolution of the extracellular metabolic composition of Aliinostoc sp. PMC 882.14 as a function of number of culture days a) under the control condition, b) under control, “higher light” and “higher temperature” conditions. Each point represents a culture replicate. The green color gradient reflects the temporal scale of the cultures.**

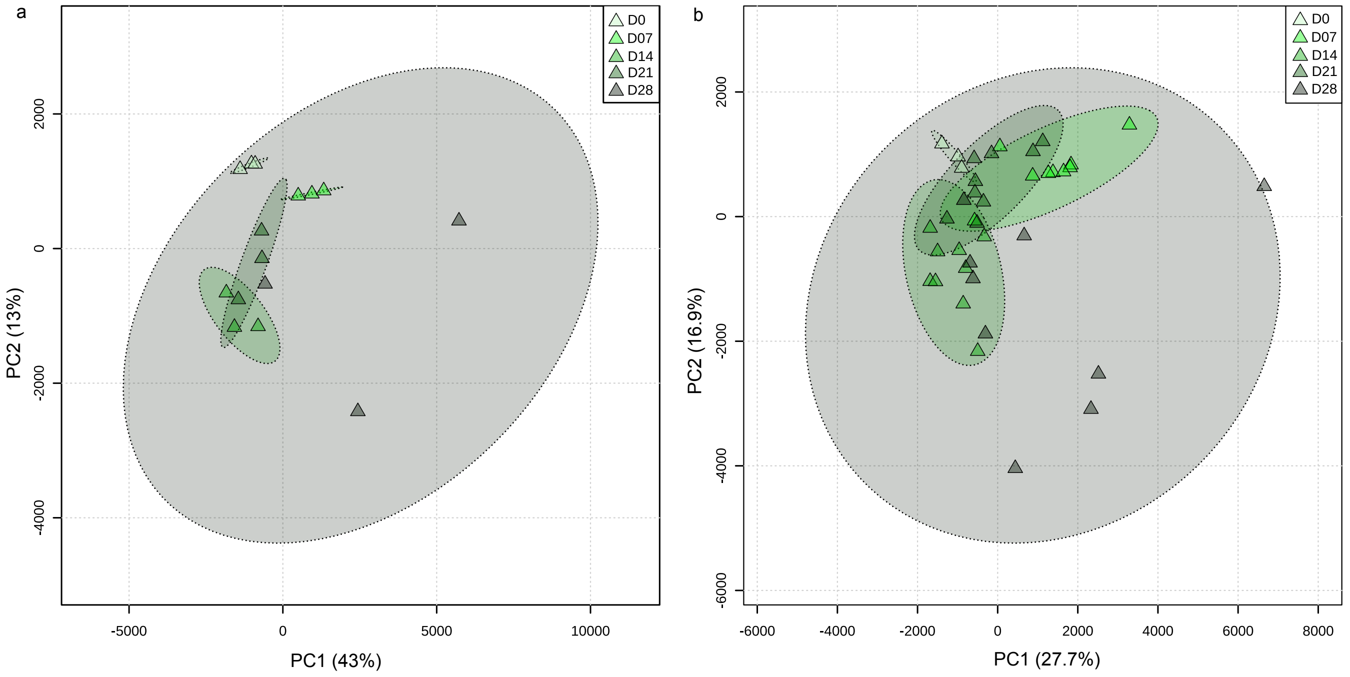

Figure S9

Principal Component Analysis (PCA) representing the evolution of the extracellular metabolic composition of *Aliinostoc* sp. PMC 882.14 as a function of culture conditions (control= grey, “higher light”= yellow and “higher temperature”= red) a) PC1 and PC2 and b) PC1 and PC3.

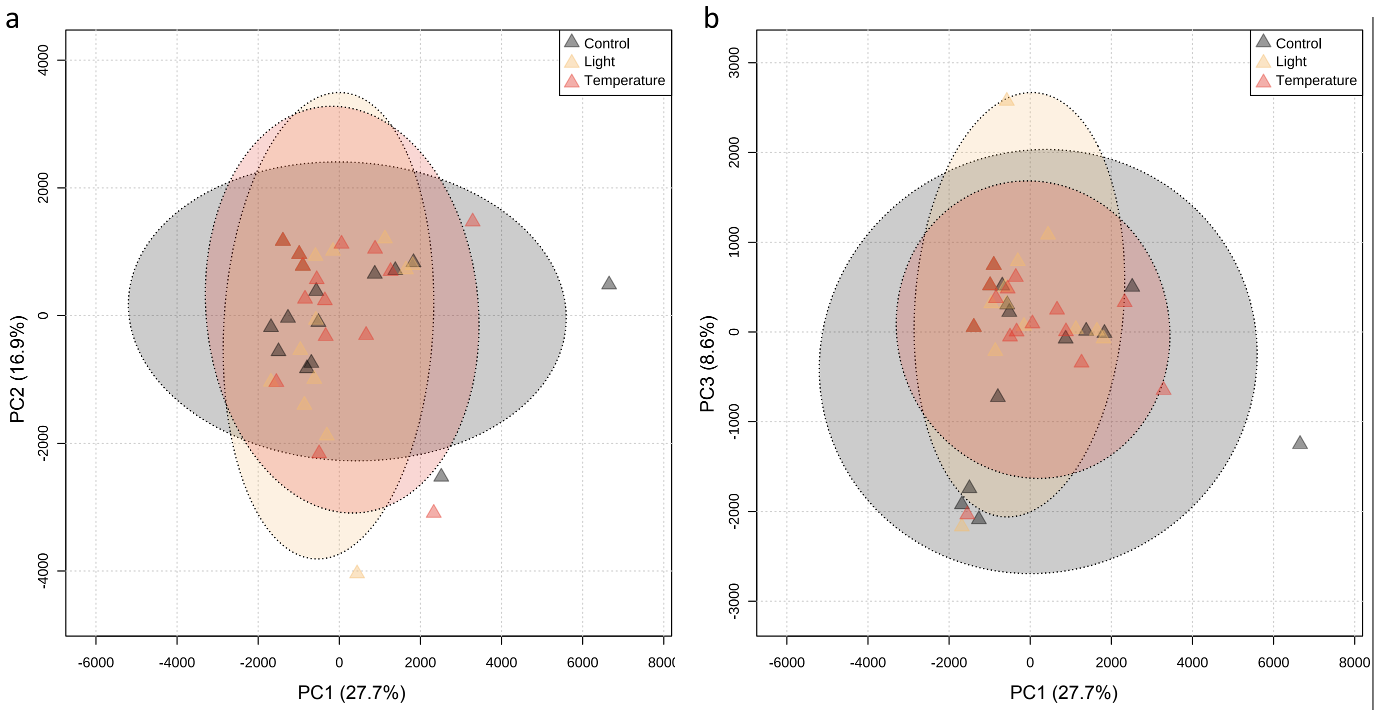

Figure S10 **PLS-DA representing the evolution of the extracellular metabolic composition of *Aliinostoc* sp. PMC 882.14 considering the days of sampling for (a) control samples only and (b) samples from the three experimental conditions and (c,d) corresponding lists of the analytes contributing the most to the sample discrimination through the culture kinetics (variable of importance in the pro-jection, VIP score >2). The red framed lines correspond to the metabolites in common with the analysis performed only on controls.**

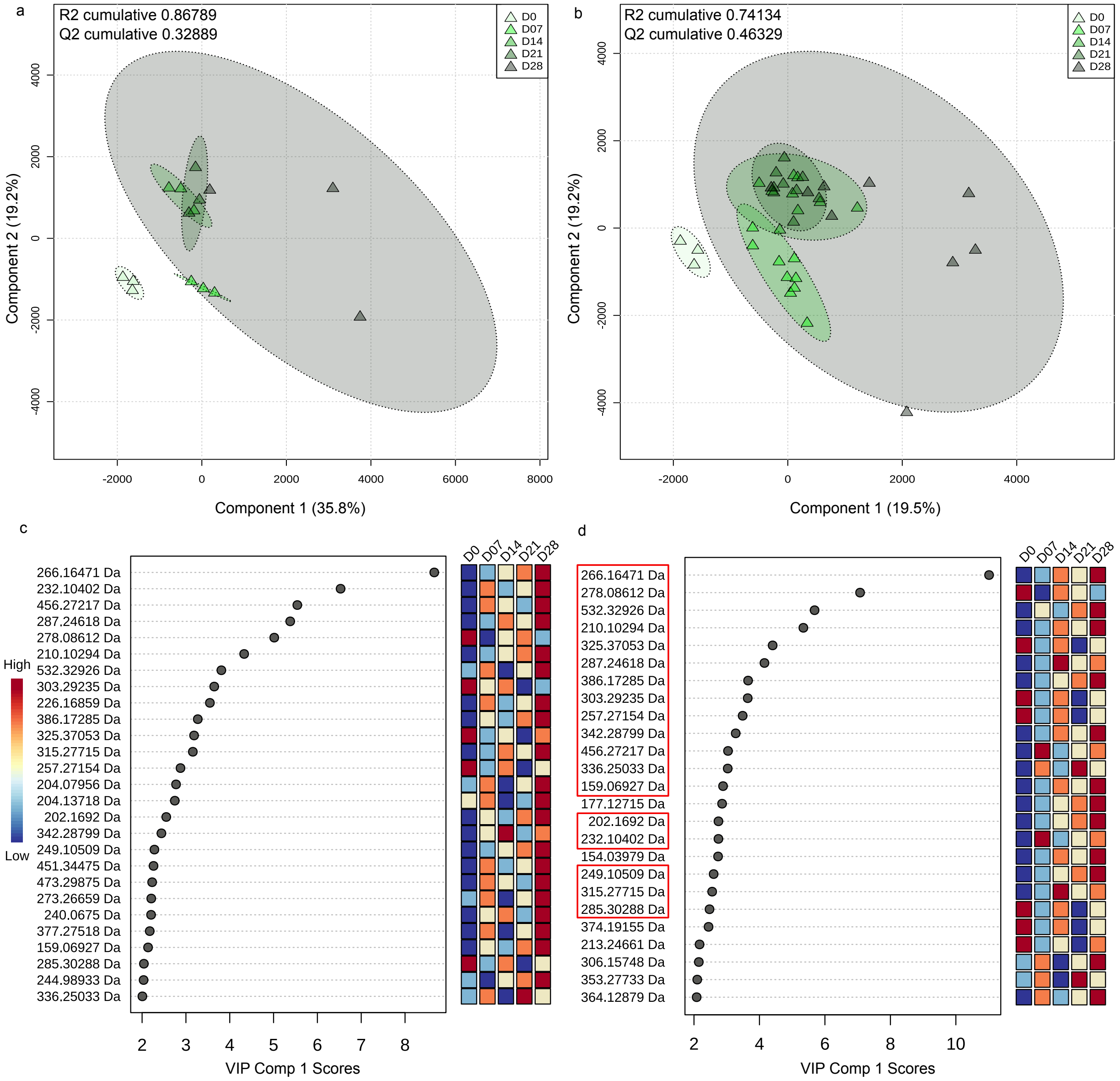

Figure S11 **Box-plots representing the 25 intracellular molecules (exhibiting VIP scores > 2; Fig S10d), which explain the most of the differences between the different days of culture considering samples from the three experimental conditions.**

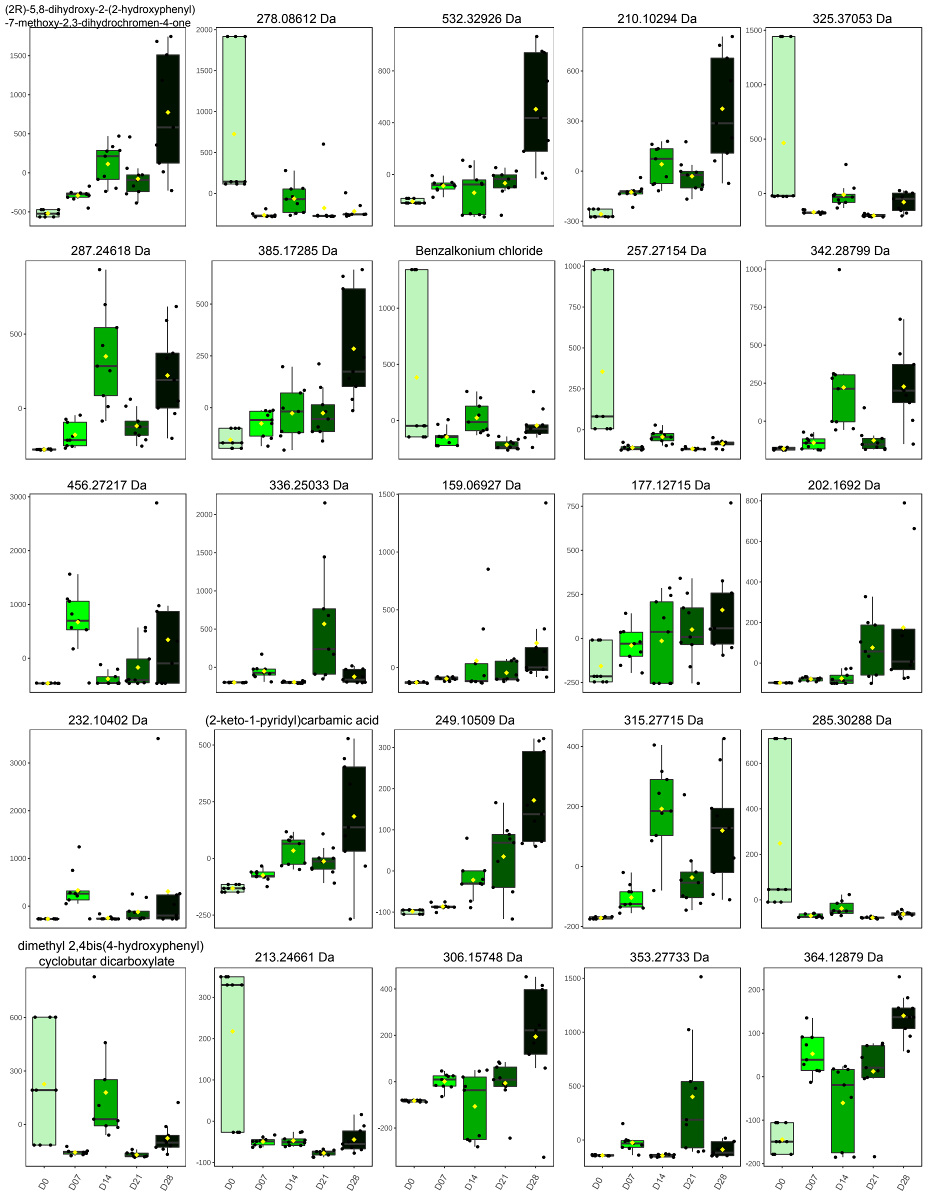

Supplement: Supplementary file 1 [file metabolites-11-00745-s001.zip › metabolites-1372065-supplementary.pdf]
